# Travis County Medical Association—Officers Elected

**Published:** 1886-09

**Authors:** 


TRAVIS COUNTY MEDICAL ASSOCIATION.

At the last meeting of the Travis County Medical Association Dr. J. Cummings was elected President; Dr. Ralph Steiner, Vice President; Dr. B. E. Hadra, Treasuerer, and Dr. Florence E. Collins, late of Chicago, but now of Austin, Secretary.

